# Sulfamethoxazole is Metabolized and Mineralized at Extremely Low Concentrations

**DOI:** 10.1021/acs.est.4c02191

**Published:** 2024-05-18

**Authors:** Ana P. Lopez Gordillo, Alba Trueba-Santiso, Juan M. Lema, Andreas Schäffer, Kilian E. C. Smith

**Affiliations:** † Institute for Environmental Research, RWTH Aachen University, Worringerweg 1, 52074 Aachen, Germany; ‡ CRETUS, Department of Chemical Engineering, Universidade de Santiago de Compostela, 15782 Santiago de Compostela, Galicia Spain; § Environmental Chemistry, Magdeburg-Stendal University of Applied Sciences, Breitscheidstraße 2, Building 6, 39114 Magdeburg, Germany

**Keywords:** antibiotic, biotransformation, biodegradation, organic micropollutants, *Microbacterium sp* BR1, ^14^C−CO_2_, threshold

## Abstract

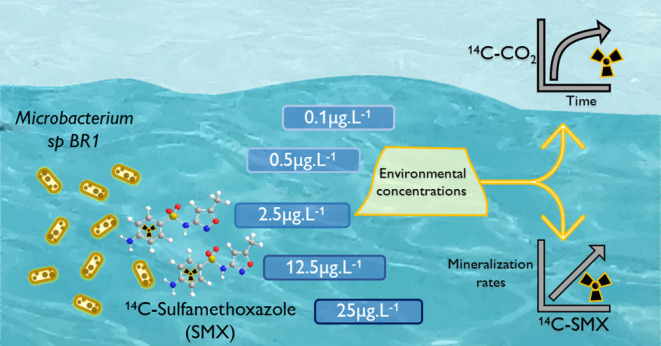

The presence of organic micropollutants in water and sediments motivates investigation of their biotransformation at environmentally low concentrations, usually in the range of μg L^–1^. Many are biotransformed by cometabolic mechanisms; however, there is scarce information concerning their direct metabolization in this concentration range. Threshold concentrations for microbial assimilation have been reported in both pure and mixed cultures from different origins. The literature suggests a range value for bacterial growth of 1–100 μg L^–1^ for isolated aerobic heterotrophs in the presence of a single substrate. We aimed to investigate, as a model case, the threshold level for sulfamethoxazole (SMX) metabolization in pure cultures of *Microbacterium* strain BR1. Previous research with this strain has covered the milligram L^–1^ range. In this study, acclimated cultures were exposed to concentrations from 0.1 to 25 μg L^–1^ of ^14^C-labeled SMX, and the ^14^C–CO_2_ produced was trapped and quantified over 24 h. Interestingly, SMX removal was rapid, with 98% removed within 2 h. In contrast, mineralization was slower, with a consistent percentage of 60.0 ± 0.7% found at all concentrations. Mineralization rates increased with rising concentrations. Therefore, this study shows that bacteria are capable of the direct metabolization of organic micropollutants at extremely low concentrations (sub μg L^–1^).

## Abstract 2

Organic micropollutant bacterial metabolism is controlled by substrate concentration. In this study *Microbacterium sp* BR1 metabolizes extremely low concentrations of sulfamethoxazole (0.1−25 μg L^−1^) when added as the only carbon source.

## 1 Introduction

In nature, oligotrophy is common in different ecosystem compartments such as groundwaters or arid soils.^1^ Accordingly, environmental bacteria have evolved strategies for the utilization of carbon substrates present at low concentrations.^2^ Threshold concentrations for microbial assimilation have been reported both in pure and mixed cultures of different origins.^3^ The literature suggests a lower range of values for bacterial growth of 1 to 100 μg L^–1^ for pure cultures of aerobic heterotrophs in the presence of a single substrate.^3^ Nevertheless, many studies have focused on “friendly substrates” such as sugars, which are very different compared to the harder-to-metabolize organic synthetic pollutants. Some reports have investigated the degradation of industrial solvents, pesticides, or pharmaceuticals within the reported concentration ranges for bacterial growth. A complete aerobic metabolization by *Burkholderia sp* strain PS14 of chlorobenzenes in soil microcosms at 100 μg L^–1^ was found. Interestingly, fructose at the same initial concentration was only incompletely and slowly metabolized.^4^ A threshold level of 30 μg L^–1^ for the pesticide atrazine with the isolated *Arthrobacter aurescens* TC1 was reported in chemostat-based experiments.^5^ In van Bergen’s study, biotransformation rate constants (h^–1^) of ten pharmaceuticals in activated sludge decreased with decreasing initial concentrations between 10 and 0.5 μg L^–1^, indicating the existence of a concentration threshold for substrate turnover.^6^

Further literature has also shown that the simultaneous utilization of easily degradable carbon sources can lower the utilization threshold for environmental chemicals fed to pure cultures (cometabolism). This was found to be the case for *Ralstonia pickettii* PKO1^7^ where higher proportions of succinate in the mixture had a positive effect on biomass production and on the time required for induction of benzene utilization. *Escherichia coli* was grown simultaneously with a fixed glucose concentration (100 mg L^–1^) and 3-phenylpropionic acid (3-PPA) added at increasing concentrations varying from 0.25–25 mg mL^–1^. The apparent threshold of extracellular 3-PPA concentrations for degradation was determined to be around 3 mg L^–1^.^8^

There is one group of ubiquitous pollutants designed to inhibit microbial growth that deserves special attention: the antibiotics. Their long-lasting contact with microorganisms leads to the appearance and dissemination of bacterial resistances. Considering that the use of antibiotics is usually the only method to treat infectious diseases, there is a risk to public health if pathogens acquire antibiotic resistance.^9,10^ A specific survival mechanism is for them to use antibiotics as carbon and energy sources, i.e., antibiotrophy.^11^ From an environmental perspective, this is particularly interesting as it protects other susceptible members of the microbiota by reducing the concentration of the antibiotic and the need for these to acquire resistance genes of their own.^12^

Among 473 compounds, including pharmaceuticals, pesticides, and industrial chemicals, sulfamethoxazole (SMX) was found to have the highest environmental risk quotient when considering occurrence and hazard estimation.^13^ For both pure strains and in wastewater treatment systems (WWT), several SMX biotransformation routes have been described, involving conjugation, oxidation, and hydrolysis reactions.^14−16^

Most of the advances done in SMX removal were achieved using wastewater (WW) communities^17^ where very high removal efficiencies have been found (>80%).^18,19^ However, SMX degradation in WW has generally been attributed to transformations not linked to growth, such as those performed by unspecific enzymes or those depending on the presence of another carbon source at higher concentration (cometabolism).^13,17,20^ There is an existing debate revolving around the ability of concentrations as low as those found typically in the environments (e.g., 10 and 8000 ng L^–1^ in WW,^16^) to induce the SMX catabolic genes. In this context, a threshold level for SMX biotransformation by ammonia oxidizing bacteria in activated sludge was found at 0.2 mg L^–1^ in a pilot-scale MBR.^13^ Simultaneous consumption of acetate and SMX (600 ng L^–1^ to 150 mg L^–1^) by a pure culture of *Achromobacter denitrificans* PR1 suggested that the energetic efficiency of the cells, favored by the addition of biogenic substrates, improved the SMX degradation rate.^21^

Ricken et al.^22−24^ obtained a pure culture of *Microbacterium sp* BR1 from WW that was capable of metabolizing SMX (and other sulfonamides) when added as the only carbon and energy source via the sulfonamide ipso-hydroxylation pathway. They described the only enzymes known to date that are involved in metabolic SMX transformation, SadABC. These are two flavin-dependent monooxygenases (AB) and a flavin reductase (C). Environmental surveys have shown that this general sulfonamide catabolic mechanism is prevalent and widely spread geographically^11,23^ as SadABC was also encoded in several other isolated bacteria from the Actinobacteria order, such as *Arthrobacter* and *Leucobacter* strains.^11^ Lab-scale SMX removal studies done so far with *Microbacterium* include concentrations in the range of mg L^–1^ (0.25–500), well above those found in environment (ng L^–1^ to μg L^–1^).^22,23,25^ Later, in a scale-up study, *Microbacterium sp* BR1 was applied for bioaugmentation in pilot-scale membrane bioreactors treating a MBR full-scale effluent and raw municipal wastewaters at temperatures <20 °C and 60–120 ng L^–1^ of SMX.^26^ SMX removal did not improve compared to the nonspiked controls, and this result was attributed to the very low SMX concentrations.

In the present study, we used *Microbacterium sp* BR1 as a degrader model to investigate the threshold and kinetics for SMX metabolism when present as the only carbon source at very low concentrations (ng L ^–1^ to μg L ^–1^), below the limit previously established in the literature of 1 μg L ^–1^.

## 2 Materials and Methods

### 2.1 Reagents and Working Solutions

Five working solutions with decreasing concentrations of radiolabeled SMX [phenyl ring-U–^14^C] (purity 99.3%, specific activity 2.954 MBq. mmol^–1^, Institute of Isotopes, Izotop) were prepared in sterile phosphate saline buffer (PBS) at pH 7.4. These solutions were spiked into the respective reactors at the beginning of the mineralization tests to obtain the desired initial test concentrations: 25, 12.5, 2.5, 0.5, and 0.1 μg L^–1^ (4.5 × 10^6^ dpm L^–1^, 2.2 × 10^6^ dpm L^–1^, 0.44 × 10^6^ dpm L^–1^, 0.085 × 10^6^ dpm L^–1^, and 0.016 × 10^6^ dpm L^–1^, respectively).

### 2.2 Bacterial Culture

*Microbacterium sp* BR1 was kindly provided by Dr. Boris Kolvenbach from the Institute for Ecopreneurship, University of Applied Sciences and Arts (Northwestern Switzerland). To activate and adapt the strain to SMX, it was cultured under sterile conditions in standard media 1 25% (Carl Roth) supplemented with 1 mM SMX (Sigma-Aldrich, 98% purity) under continuous agitation of 140 rpm and at 28 °C until reaching an early stationary growth phase with an optical density (OD_600_) of 1.4. The produced biomass was centrifuged at 7000*g* and 4 °C for 20 min and then washed twice with cold NaCl (0.85%). Bacterial pellets were resuspended in a preserving solution (NaCl (0.85%) and glycerol (20%)) before their homogeneous distribution in aliquots, followed by storage at −80 °C. Aliquots were thawed prior to their use in the mineralization tests.

### 2.3 Mineralization Tests

The turnover of ^14^C-SMX into ^14^C–CO_2_ was monitored as an indicator of mineralization. Batch experiments were carried out with five initial concentrations of ^14^C-SMX: 25, 12.5, 2.5, 0.5, and 0.1 μg L^–1^. Amber glass bottles of 50 mL served as the reactors and initially contained 26 mL of PBS. Mineralization tests were started after adding 1 mL of the corresponding SMX working solution and 1 mL of thawed *Microbacterium sp* BR1 as the inoculum to each bottle. An insert preloaded with 1 M KOH was then added to capture any produced ^14^C–CO_2_, and the bottles were closed until sampling. The estimated OD in each reactor at the start of the experiment was 0.05. In addition, serial dilutions of the inoculum were streaked on agar plates to determine the colony forming units (CFU) at the beginning of the test (209.8 × 10^6^ CFU mL^–1^ ± 0.34). The abiotic controls consisted of bottles without bacteria. All experiments were performed under sterile conditions at 22 °C in the dark and under horizontal agitation (140 rpm).

Four sampling points were distributed over the 24 h of each test (2, 4, 8, and 24 h). At each time point, triplicate reactor bottles were sacrificed to analyze the KOH trapping solution and 1 mL of the reaction medium. The KOH and 1 mL medium samples were mixed with 2 mL of Ultima Gold XR scintillation cocktail (PerkinElmer, Germany), and the radioactivity was measured using a liquid scintillation counter (LSC, Hidex 600/300 SL, Finland). The remnant of the reaction medium was stored at −26 °C.

### 2.4 Determination of the Parent SMX Concentration

In order to determine the nature of the radioactivity remaining in the reaction media, i.e., whether this was nonmetabolized parent SMX, frozen samples from the reaction media were subject to liquid–liquid extraction (LLE) and radioanalysis. For the 25 μg L^–1^ concentration, all time points were extracted to obtain a time series of the SMX depletion. For 12.5 and 2.5 μg L^–1^, the final time point was analyzed to determine residual SMX at test completion. Approximately 20 mL of reaction media were defrosted and acidified to pH 2 with 140 μL of 15% HCl. Then 3 mL of a mixture of ethyl acetate and n-hexane (5:1) was added to the media for the first extraction step. After 40 min of horizontal shaking at 140 rpm, the samples were frozen to facilitate the separation of the solvent phase, which was collected in a glass vial prerinsed with methanol. For the second and third extraction steps, 3 mL of a mixture of ethyl acetate and *n*-hexane (3:1) was added, and the shaking-freezing cycle was repeated. The three solvent phases were pooled in the same glass vial and evaporated until dry under a gentle N_2_ stream. Then 150 μL of methanol was added and a brief sonication was used to resuspend the sample before transferring to a high-performance liquid chromatography (HPLC) vial. This resuspension step was repeated three more times: once more with methanol and then twice with MiliQ water. The resuspended fractions were pooled in the HPLC vial, resulting in 600 μL of methanol:water (1:1). The resulting sample was injected into an HPLC (Agilent Infinity II 1260, Agilent) coupled to a radioactive detector (Raytest Ramona liquid cell detector, Elysia, Belgium). Qualitative detection of ^14^C-SMX was corroborated with the signal of the ^14^C-SMX standard. The mobile phases consisted of MiliQ water with 0.1% formic acid (A) and methanol with 0.1% formic acid (B) in a gradient with a flow rate of 0.9 mL min^–1^. The eluent was collected in vials at intervals of 0.5 min and mixed with Ultima Gold XR scintillation cocktail (PerkinElmer, Germany). Eluent′s radioactivity was measured using LSC to quantify changes in SMX concentration over the course of test. The end concentrations of SMX for the 0.5 and 0.1 μg L^–1^ concentrations could not be measured using radio HPLC as they were below detection limits.

## 3 Results and Discussion

### 3.1 Mineralization Thresholds

**Figure 1:**
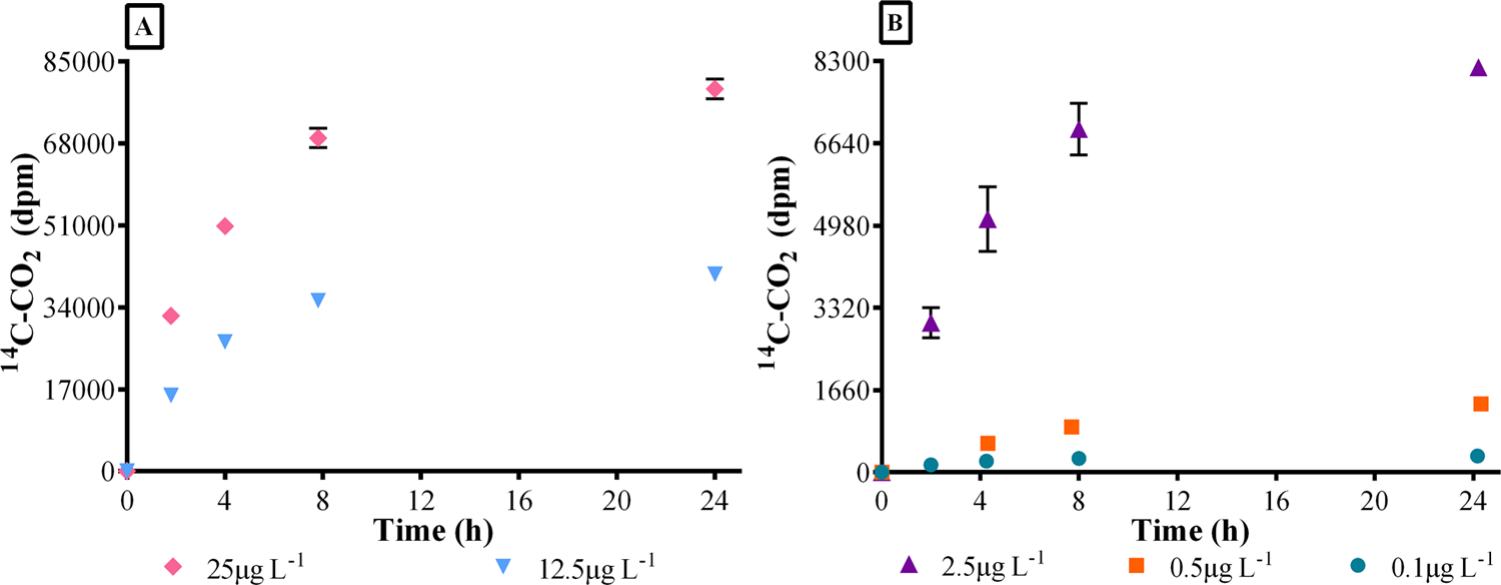
Cumulative production of ^14^C–CO_2_ for the five SMX mineralization tests: for clarity, mineralization kinetics are distributed in section (A) (25 and 12.5 μg L^–1^) and (B) (2.5, 0.5, and 0.1 μg L^–1^). Depicted values are means of triplicate with their standard deviations. dpm is decays per minute.

*Microbacterium sp* BR1 metabolized SMX in all of the conducted tests. In Figure 1A,B, it can be observed that mineralization occurred in the five tests and that produced ^14^C–CO_2_ was proportional to the concentrations of SMX. ^14^C–CO_2_ production in the test of 0.5 μg L^–1^ progressed slightly slower than in the other tests due to a possible overspill of KOH traces into the reaction media at the beginning of the experiment.

This observation that ^14^C–CO_2_ was produced at all of the different test concentrations shows for the first time that a bacterial strain is not only capable of biotransforming but also mineralizing a micropollutant at concentrations 10 times lower than the threshold of 1 μg L^–1^ reported for the utilization of single substrates by pure strains.^3^ In this context, it is probable that any existing SMX mineralization threshold of *Microbacterium sp* BR1 lies below 0.1 μg L^–1^. However, the investigation of a threshold at even lower concentrations was constrained by analytical detection limits.

One reason why any threshold of *Microbacterium sp* BR1 for SMX degradation could exist at such low concentrations is due to the successful acclimation of the bacteria, leading to the expression of enzymes (SadABC) involved in the SMX catabolic pathway.^23^ Furthermore, changes in the bacterial morphology (e.g., miniaturization) may serve to increase the surface-to-volume ratio and improve substrate uptake at low concentrations.^27,28^ This is in accordance with Poursat et al.^29^ who stated that the preadaptation of a bacterial strain to a pollutant promotes its biotransformation and therefore lowers the threshold for its utilization. Bacterial acclimation is also likely in WWTP, where a background concentration of micropollutants present in the activated sludge may favor the adaptation of the microbial community with a higher biotransformation potential.^6,29^ In this scenario, bacterial acclimation would mostly occur at low concentrations (from ng L^–1^ to μg L^–1^). Nevertheless, micropollutant point sources and seasonal events such as droughts lead to peak load scenarios that might also result in bacterial acclimation at concentrations close to those applied in our study (mg L^–1^). Moreover, it has been reported that the expression of transporters with affinity for a substrate and the corresponding catabolic enzymes can occur in carbon/energy limited environments (even in the absence of the substrate), where bacteria usually have a slow growth rate.^3^ This aspect was also considered in this study when harvesting acclimated strain BR1 during the early stationary phase.

### 3.2 Mineralization and Biotransformation Rates

The mineralization rates (Table 1) show the influence of the test concentrations on the production of ^14^C–CO_2_. It can be observed that the fastest ^14^C–CO_2_ production (0.0314 μg (L h 10^9^CFU)^−1^) corresponds to the 25 μg L^–1^ test, and the slowest rate of 0.00013 μg (L h 10^9^CFU)^−1^ to the 0.1 μg L^–1^ test. The 240-fold difference in rates coincides with the 250-fold gap between the extremes in the tested concentrations.

**Table 1:** Mineralization Rates of *Microbacterium sp* BR1 at Environmental Concentrations. SMX Served as the Only Energy and Carbon Source

test concentration	mineralization rate
μg L^–1^	μg (L h 10^9^CFU)^−1^
0.1	1.30 × 10^–4^
0.5	3.33 × 10^–4^
2.5	2.60 × 10^–3^
12.5	1.56 × 10^–2^
25	3.14 × 10^–2^

**Figure 2:**
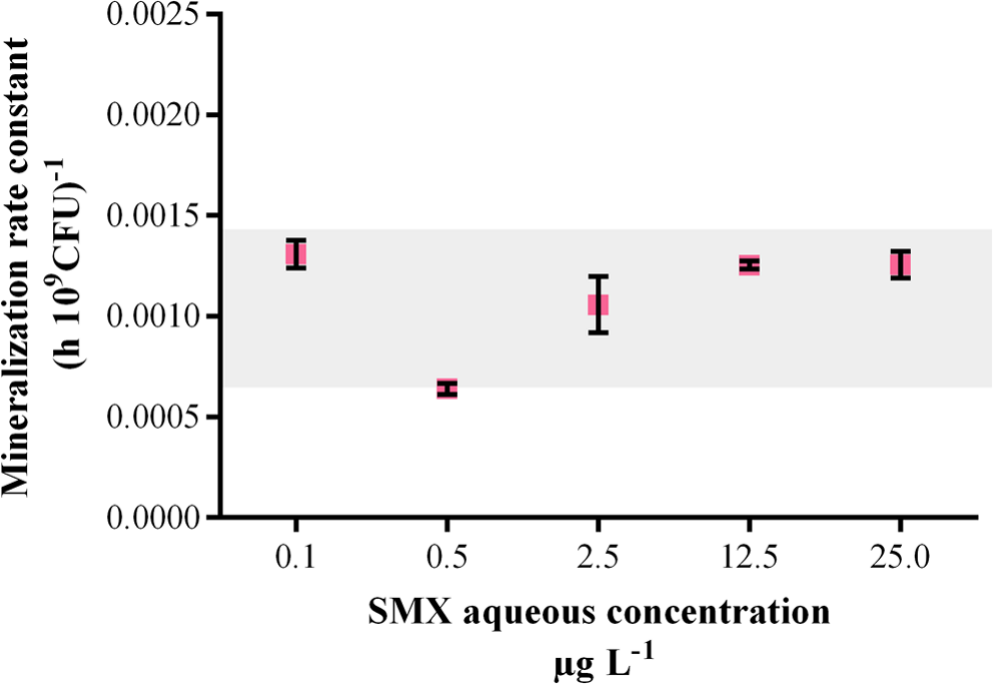
Mineralization rate constants from the five biotransformation tests with increasing initial SMX concentrations. The standard deviation is shown for the experiments that were performed in triplicate. The values were normalized by the bacterial density and the initial SMX concentration.

Mineralization rates of the five SMX initial test concentrations were considered to follow first order kinetics, showing a rise in the rates that was proportional to the factor increase in the initial concentrations (Table 1). Van Bergen et al.^6^ mentioned that this behavior is expected at low concentrations of micropollutants in wastewater due to the initial linear range of the Michaelis–Menten relationship between reaction rate and the substrate concentration. A decrease in the corresponding first order rate constants (h^–1^) with decreasing substrate concentrations would indicate a concentration threshold for substrate turnover. However, in our investigation, mineralization rate constants were within the same range (Figure 2) at all tested submicromolar SXM concentrations. This suggests that the pure strain *Microbacterium sp* BR1 is capable of metabolizing concentrations down to 0.1 μg L^–1^ of SMX, which is in the same low range of reported biotransformation threshold concentrations of several other micropollutants: 0.1–3 μg L^–1^ for 2,6-dichlorobenzamide, 0.4–1.8 μg L^–1^ for acetaminophen and metformin, 10 and 32 μg L^–1^ for the pesticide atrazine.^5,6,30,31^

Most SMX removal assays focus on either the fraction of SMX that is depleted or mineralized, without simultaneously considering both the biotransformation and mineralization rates.^14,32^ However, the inclusion of both of these rates allows a better understanding of the kinetics occurring at both extremes of the catabolic pathway. Biotransformation rates (i.e., depletion of the parent SMX) were calculated for other investigations conducted with pure strains after their values were converted to common units. These are displayed in Table 2, together with the SMX biotransformation rate determined for the 25 μg L^–1^ test concentration from this study.

**Table 2:** SMX Biotransformation Rates and Rate Constants from Pure Bacterial Strains in Mineral Salt Media under Aerobic Conditions^a^

bacterial strain (s)	SMX initial concentration mg L^–1^	incubation period days	biotransformation rate mg (L h)^−1^	biotransformation rate constant h^–1^	refs
*Microbacterium sp* BR1	0.025	1	0.006	0.235	in this study
*Microbacterium sp.* SMXB24 *and sp.* SMX348 (BR1)	10	10	0.05	0.005	(25)
*Microbacterium sp* BR1	25.4	0.83	44.07	1.73	(22)
*Pseudomonas sp.* SMX330, 331, 344, 345	10	10	0.05	0.005	(25)
*Pseudomonas sp.* SMX321	10	10	0.07	0.007	(25)
*A. denitrificans* PR1	150	14	18.51	0.12	(16)

a SMX served as the only carbon, energy, and nitrogen source. For comparison purposes, the content is limited to the studies where a biotransformation rate was provided or where the rate constant could be calculated. However, not all of the studies applied a normalization by bacterial density.^22^

Considering the *Microbacterium sp* BR1 biotransformation rates from all studies including this one, at initial concentrations of 0.025, 10, and 25.4 mg L^–1^, these do not appear to follow the proportional increase with concentrations as was observed for the mineralization rates in this study (compare Tables 2 and 1). Although there is a trend for an increase in the biotransformation rate with the concentration, a direct comparison is not possible between all of the studies due to the lack of normalization by cell density. After accounting for the effect of the initial concentrations via normalization, the values of the corresponding rate constants range over about 3 orders of magnitude: 0.235 h^–1^ (this study) compared to 0.005 and 1.73 h^–1^ in other studies. This discrepancy could indicate that strain BR1 has a distinct biotransformation capacity. It is likely that the acclimation conditions and the growth stage of the inoculum utilized in each study play a role. Further comparison of the biotransformation rate constants from tests conducted with the same inocula might be suitable for investigating this.

Differences between biotransformation rate constants at mg L^–1^ and (sub) μg L^–1^ concentrations might indicate different kinetic regimes at low and high concentrations.^33^ In the range of ng L^–1^ to μg L^–1^, after several weeks of steady state concentrations, the enzymatic activity could be downregulated by mass transfer constraints.^6^ Conversely, the increase in the rate constant at mg L^–1^ levels suggests a higher biotransformation capacity, but this may decrease when the concentration has inhibitory or toxic effects on the bacteria.^34^ Usually, this inhibition occurs well above typical environmental concentrations (i.e., in the μM or μg L^–1^ range).^6^ For *Microbacterium sp* BR1, the highest tolerated concentration without toxic or inhibitory effects has been reported to be 127 mg L^–1.^^14^^14^ In the case of *Pseudomonas silesiensis* F6a, inhibition of SMX biotransformation was observed at 80 mg L^–1^,^35^ and for most SMX degraders, the highest tolerated or tested concentration was below 200 mg L^–1^.^11^

Directly comparing biotransformation rate constants (h^–1^) between different bacterial strains is difficult due to the different responses of the strain to the pollutant concentration. As an example, *A. denitrificansPR1* showed a lower rate at 150 mg SMX L^–1^ compared to that of *Microbacterium sp* BR1 incubated with 25.4 mg SMX L^–1^ (see Table 2). However, it has also been reported that the biotransformation rate of the former may decrease as SMX concentrations increase into the mg L^–1^ range.^16^

Comparing biotransformation and mineralization rates under the same conditions facilitates the investigation of limiting concentrations at different steps of the metabolic pathway. In this study, at the 25 μg L^–1^ test concentration, the biotransformation rate constant was calculated to be 0.235 (h 10^9^CFU)^−1^ (Table 2). In contrast, the mineralization rate constant was markedly lower at 1.26 × 10^–3^ (h 10^9^CFU)^−1^ (see Figure 2). This indicates that the initial biotransformation step for parent SMX was much faster than its complete mineralization. Nevertheless, these rates do not reveal the presence of a threshold concentration.

The rates shown in Table 2 may vary under cometabolic conditions. This refers to the biotransformation of SMX in the presence of an extra carbon source and nutrients. An increase in biotransformation rates from 1 to 2.5 mg (L d)^−1^ has been reported after adding an extra source of carbon and nitrogen.^25^ Moreover, the mineralization and biotransformation rates of pure cultures may be enhanced in cocultures. This was previously described when the SMX biotransformation rate of *A. denitrificans* PR1 had a ca. 2-fold increase when mixed with three additional pure strains: *Ochrobactrum intermedium* PR2, *Pseudoxanthomonas indica* PR3, and *Agromyces soli* PR4.^16^ These three strains did not contribute directly to SMX biotransformation, but they appear to have released cofactors that enhanced the strain PR1 biotransformation rate. This was concluded since a similar rate was obtained in tests that solely included strain PR1 supplemented with amino acids, vitamins, and nitrogen bases.

### 3.3 Fraction of SMX Mineralized

**Figure 3:**
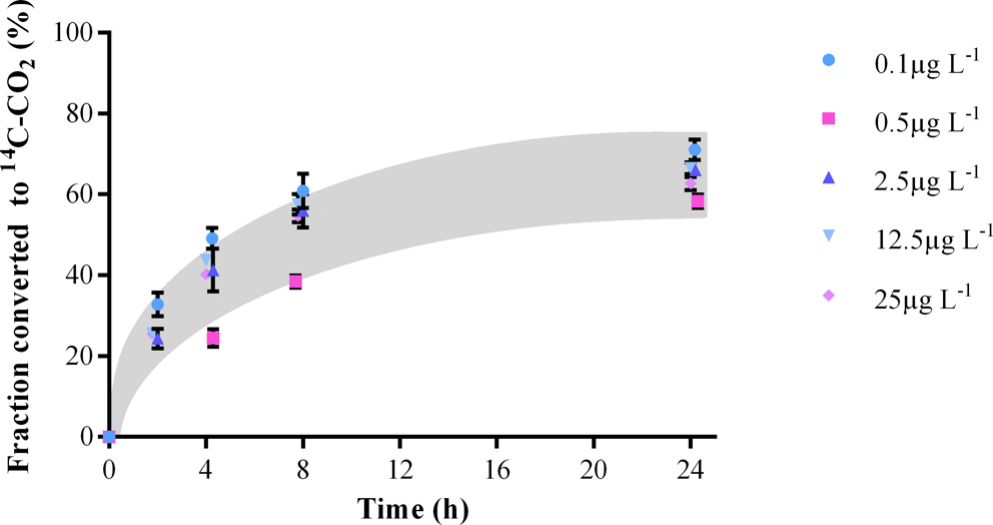
Evolution of SMX mineralization at the five initial concentrations. Values are the means of triplicates with their respective standard deviations.

The total fraction of ^14^C–CO_2_ produced was similar for each tested SMX concentration: *Microbacterium sp* BR1 mineralized around 60 ± 0.67% of the initial SMX concentration (Figure 3). ^14^C–CO_2_ could be measured already after 2 h of incubation, which indicates that any lag phase was avoided through the preadaptation of the strain to SMX in our study. After about 8 h, 40–60% of the added ^14^C-SMX had been converted to ^14^C–CO_2_ and this was followed by a plateau in the mineralization rate until the end of the experiment. This agrees with previous results,^14^ where *Microbacterium sp* BR1 was incubated with SMX at 126.63 mg L^–1^ and reached a mineralization of 44% after 16 days followed by a mineralization plateau. In another study, SMX was mineralized up to 48% after 5 days of incubation with *Microbacterium sp* BR1.^23^ Similar mineralization for SMX has been reported for *Rhodococcus sp* BR2 and *Achromobacter sp* BR3 (22–44%),^23^ whereas the *Acinetobacter sp* W1 strain mineralized SMX to a much higher extent (95 to 100%).^32^ Such a high mineralization value could be explained as *Acinetobacter sp* W1 follows a metabolic pathway with more biodegradable metabolites than the recalcitrant 3-amine-5-methylisoxazole (3A5MI) reported for *Microbacterium sp* BR1.

**Figure 4:**
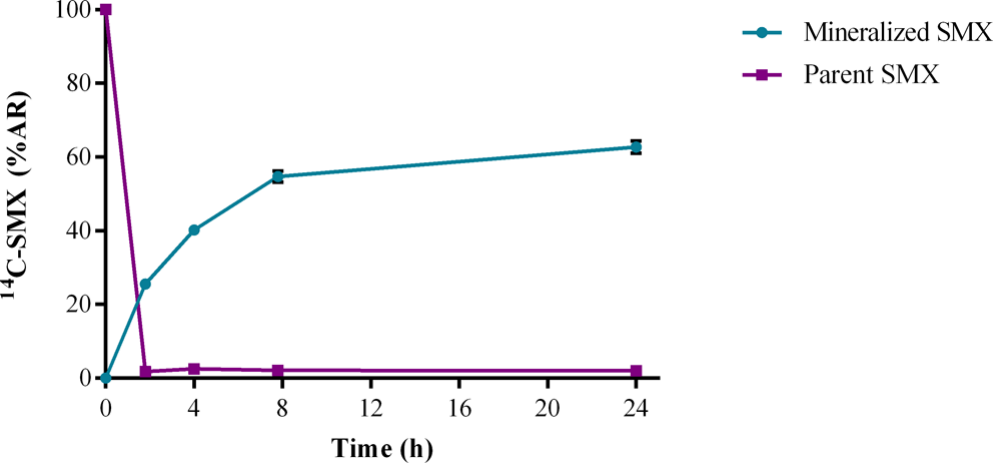
Evolution of ^14^C-SMX mineralization coupled to its depletion in the reaction medium for the test concentration of 25 μg L^–1^. AR = applied radioactivity.

The mineralized fractions of approximately 60% obtained in Figure 3 fit with the reported range of mineralization occurring after the complete destruction of a chemical (50–80%).^33^ In this sense, monitoring both biotransformation and mineralization of SMX in this study allowed the extent of parent SMX remaining in the reaction media to be determined, and this tallied with an almost complete removal of parent SMX (98% after 24 h; see Figure 4). A similar biotransformation behavior was observed for the test concentrations of 12.5 and 2.5 μg L^–1^, where the fraction of parent SMX was determined only at 24 h and was 2 and 3.2%, respectively. Another investigation that monitored the biotransformation-mineralization balance found that approximately 80% of the parent SMX was removed and only 26% was mineralized after 8 h of incubation at 60 mg L^–1^ with a mixed culture derived from activated sludge.^36^ In this case, the low mineralized fraction was attributed to incomplete mineralization of the formed byproducts. According to this, Figure 4 explains the fate of 62% of the initial parent SMX, whereas the missing radioactivity (35%) possibly is distributed as SMX metabolites and biomolecules. However, to confirm this, further studies of biotransformation products and metabolic pathways are necessary.

The observed decrease in the mineralization rate after 8 h is a delayed effect caused by the drastic depletion of parent SMX during the first stage of the experiment. However, the delay in this decreased CO_2_ production compared to the rapid disappearance of the parent SMX is expected since multiple catabolic steps must occur from SMX primary biotransformation until its full mineralization. The biotransformation rates of each intermediate catabolic step influence the duration of the observed delay.

Despite a nearly complete depletion of SMX after 2 h, the plateau of mineralization only commenced after 8 h and the mineralized fraction increased by 11% after 24 h. This and the lack of any trend in the mineralization rate constants support the idea that a threshold for mineralization was not found in this study. Extending the experiment to 48 h for the 0.5 μg L^–1^ test concentration resulted in an additional increase of only 5% in the CO_2_ fraction and a final mineralized fraction of 63% which was similar to the mineralization trends observed for the other test concentrations (Figure S2). This confirms that the catabolic machinery of *Microbacterium sp* BR1 was still active until at least 48 h regardless of the observed SMX depletion and thus that the mineralization plateau observed at 8 h was not caused by enzymatic limitations. Further mineralization tests with a longer duration were out of the scope of this study since strain BR1 already showed an almost complete biotransformation and mineralization response in the first 24 h of the test. Nonetheless, it is possible that bacterial activity would differ in long-term incubations at steady low concentrations, where the cells could have sufficient time to change their morphology and down regulate their enzymatic kinetics to counteract mass transfer limitations. This would be an important direction for future studies in this area.

Degradation kinetics will vary if bacterial growth occurs,^37^ with a faster response during the exponential growth phase than during the stationary phase when nutrients become scarce.^38^ In our study, bacterial density was determined only at the beginning of the test. However, an increase in turbidity in the reaction media and an increase of bacterial pellet size were observed over time in analogous nonradioactive SMX tests (data not shown). As the growth of *Microbacterium sp* BR1 from the SMX aniline moiety has been previously reported,^22^ it is possible that the SMX biotransformation and mineralization rates observed during the 24 h of test were influenced by biomass growth^39^ on the SMX. Any potential growth caused by the carbon released during cell lysis was not assessed.

Figure 4 shows that after 8 h, the fraction of parent SMX remained low at around 2%. Therefore, 100% removal of the parent SMX was not achieved. The 98% SMX removal in our study likely reflects the effect of bacterial acclimation to mM levels. The reason for the low residual parent SMX of 2–3% measured for the three highest concentrations requires further study. It should be clarified whether these are some sort of common residual threshold or represent a pool of parent SMX that is not or is only slowly bioavailable to the degrading cells (e.g., due to sorption). In general, a complete removal of sulfonamides is not achieved by conventional activated sludge.^17^ SMX is typically removed to levels between 0 and 90% of the initials in WWTP.^40−43^ It has been reported that a sequential degradation with diverse bacterial strains may become necessary when the produced metabolites become recalcitrant.^33^ In this regard, although a complete degradation of SMX was reported for *Microbacterium sp* BR1 at 25 mg L^–1^,^22^ it was also reported that the formation of the metabolite 3A5MI can delay SMX biotransformation by 28%.^16^

**Figure 5:**
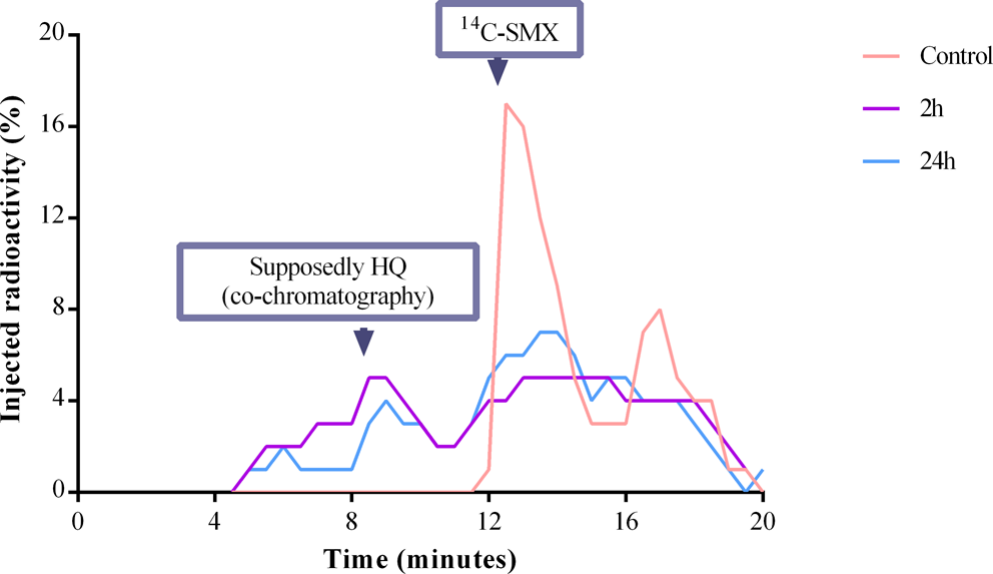
LSC chromatogram obtained from the test 25 μg L^–1^ for the identification of parent ^14^C-SMX and detection of a ^14^C-labeled byproduct. The three different series show the radioactive signals of the samples after their chromatographic separation. The signal between 8 and 10.5 min corresponds to the suspected HQ metabolite, based on a similar RT of the nonradioactive standard. The parent ^14^C-SMX is located within 12 and 15.5 mins.

Based on the radio-HPLC analyses of the reaction media at the test concentration of 25 μg L^–1^, it was possible to detect an additional radioactive signal besides that of ^14^C-SMX (see Figure 5). In the solvent extract of the biotic control containing autoclaved bacteria, the main radioactive peak could be attributed to the parent SMX based on injection of a pure standard. However, this was followed by a smaller secondary peak with a later retention time (RT) that was not seen for the pure standard. This was due to a chromatographic artifact because of coextractives in the autoclaved medium, leading to band broadening and poor separation of the SMX. In the 2 and 24 h samples from the degradation experiments, the parent SMX peak had disappeared. Furthermore, there is initial evidence of an additional peak with an earlier RT than the SMX that decreases over time. After comparing the RT of this peak to those of the nonradioactive metabolite standards 3A5MI, 1,2,4-trihydroxybenzene (THB) and hydroquinone (HQ), we suggest that the signal corresponds to the latter. Further studies that include LC-MS analytics are needed to identify the metabolite. In any case, HQ has not been reported to interfere with the catabolism of SMX by *Microbacterium sp* BR1.

In the present study, we have shown how *Microbacterium sp* BR1 is a very sensitive antibiotroph that can mineralize SMX when this is added as the only carbon source. This occurs even at the very low concentrations that are typically observed in environmental media, including wastewater. Furthermore, differences in the biotransformation versus mineralization rates were ascertained, providing further insights into the disappearance of the parent SMX relative to its complete conversion into inorganic forms. The latter is the desired end point for the biological treatment of micropollutants^36^ to avoid possible toxicity due to intermediate metabolites. Finally, the results show that BR1 is able to degrade SMX at values below the previously suggested literature threshold of 1 μg L^–1^ for pure cultures with single substrates. This is also different from what has been reported previously for mixed communities degrading SMX. For instance, a much higher threshold level of 0.2 mg L^–1^ was suggested in activated sludge, with the high transformation rates previously reported for WWTPs generally being attributed to cometabolism. Isolated bacteria with different SMX biotransformation metabolism (*Microbacterium* and *Achromobacter)* that were reintroduced into the real environments (WWTP) were not successful in either enhancing SMX removal or surviving in the populations. The low energy yield of the SMX catabolism in this range of values could explain why *Microbacterium* and *Achromobacter* growth was not sustained over time in these bioaugmentation tests and this required periodical reinoculations. A competition for nutrients and energy-rich substrates between the antibiotrophs and native microorganisms likely hampers any successful bioaugmentation.

This study broadens our understanding and characterization of micropollutant biodegradation by pure cultures in terms of biotransformation and mineralization rates, limitations for a complete SMX removal, and the impact of bacterial acclimation. This is relevant for a better understanding of how the concentration of a micropollutant affects its incomplete removal in WWTP effluents. However, the results also indicate that it is necessary to explore additional factors that might have a greater impact on the complete depletion of micropollutants in the WWTP, such as the composition of the mixed microbial communities or the presence of diverse carbon sources. A better insight on this would bring us closer to further optimizing strategies such as bioaugmentation and selecting conditions that boost the bacterial degradation of micropollutants.

Future investigation of threshold concentrations should entail discussions about the focus on mass transfer limitations or physiological limitations.^31,44^ In the present study, the biotransformation and mineralization results indicate that SMX uptake into the bacteria and enzymatic activity were not restricted. Isotope fractionation techniques would help to investigate the influence of mass transfer on the rate constants. Additionally, analysis of the proteome profile may help to confirm continued enzymatic activity.^5^ A comparative assessment between organisms adapted to mg L^–1^ and to low environmental concentrations (∼10 μg L^–1^) is therefore necessary to clarify the impact of the acclimation on the residual SMX levels. Further search into byproducts produced at the ng L^–1^ to μg L^–1^ level could help to understand if the small amounts of parent SMX are related to 3A5MI inhibition, limiting its complete biotransformation and mineralization.

## Supporting Information Available

Additional experimental material and method; including photographs of agar plates for colony counting (PDF)
