# Supplementary material for: Sulfamethoxazole is Metabolized and Mineralized at Extremely Low Concentrations
Source: Environ Sci Technol. 2024 May 18;58(22):9723–30. doi: 10.1021/acs.est.4c02191 (PMC11155234; doi:10.1021/acs.est.4c02191)
Supplement: Supplementary file 1 — es4c02191_si_001.pdf [file es4c02191_si_001.pdf]

Supplementary Information

Sulfamethoxazole is metabolized and mineralized at extremely low concentrations

Ana P. Lopez Gordillo [1,2], Alba Trueba-Santiso [2*], Juan M. Lema [2], Andreas Schäffer [1] and Kilian E.C. Smith [3]

[1]Institute for Environmental Research, RWTH Aachen University, Worringerweg 1, 52074 Aachen, Germany

[2]CRETUS, Department of Chemical Engineering, Universidade de Santiago de Compostela, 15782 Santiago de Compostela, Galicia, Spain.

[3]Environmental Chemistry, Magdeburg-Stendal University of Applied Sciences, Breitscheidstraße 2, Building 6, 39114 Magdeburg, Germany

[*] Corresponding author. E-mail address: albamaria.trueba@usc.es. Tel.: +34 881816020.

Supplementary data contains:

4 Pages

1 Text

2 Tables

2 Figures

Table S1. Composition of Phosphate Buffer Solution (PBS) 1X used in the mineralization tests.*

Reagent	mM	g L^{-1}
NaCl	137	8.006
KCl	2.7	0.201
Na_2HPO_4	10	1.419
KH_2PO_4	1.8	0.244

*Mili Q water was used to prepare the buffer. After the salts were dissolved, the pH was adjusted to 7.4 and the buffer autoclaved at 121°C for 15 minutes.

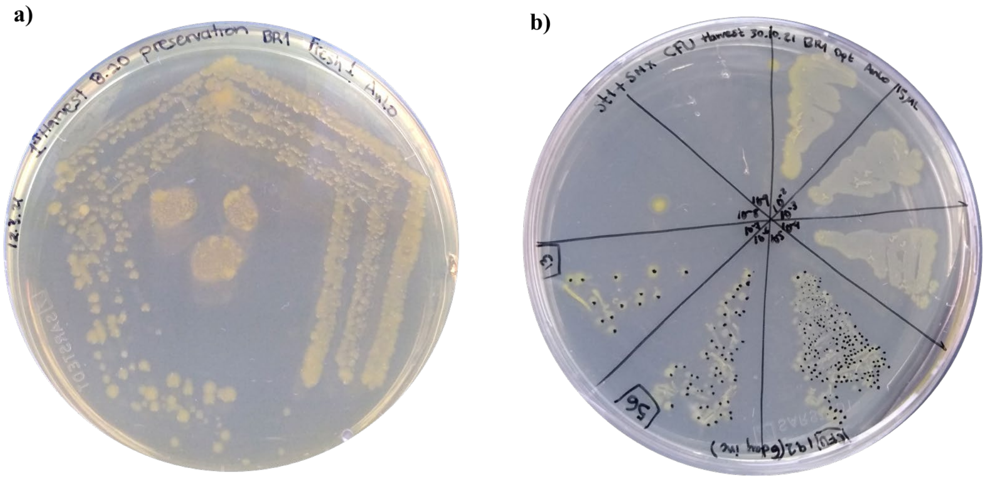

Figure S1. Example of agar plates used for a) the contamination checks of the strain BR1 and b) for quantification of bacterial density (CFU) of the inoculum used for mineralization tests. The endo agar design accommodating multiple serial dilutions per plate was used to enumerate the colonies of *Microbacterium sp BR1* after 24h of incubation at 30°C.

Text S1. Methodology utilized for the calculation of the biotransformation and mineralization rates. The following steps were applied for each sampling point of the pertinent test concentration: the averages of the radioactivity contained as ^{14}C-CO_2 in the KOH or as residual ^{14}C-SMX in the reaction media, were transformed to μg L^{-1} units using the specific activity of the ^{14}C-SMX applied to the experiments (177161.26 dpms.μg^{-1}). The obtained values of mineralization were fit to a one-phase association model using GraphPad Prism 6 and the values for biotransformation were fit to a single first order decay model using the Computer Assisted Kinetic Evaluation (CAKE) program. With this, the rate constants (h^{-1}) were obtained, all with a goodness of fit r^2 >0.9. Bacterial density plays a role in the calculated conversion rate inasmuch as this will increase in the presence of more acclimated bacteria with a stock of enzymes suited for SMX catabolism. Thus, the rate constants were normalized by the bacterial density inoculated at the beginning of the tests (h 10^9CFU$)^{-1}$ (see Table S2). Normalized rate constants were multiplied by the test concentration to calculate the corresponding mineralization or biotransformation rates μg (L h 10^9CFU$)^{-1}$. The normalized rates correspond to the values shown in the Table 1, Figure 2 and Table 2.

Table S2. Summary of data used to calculate normalized rates and rate constants from the rates obtained with model fitting.

Test concentration *μg L^{-1}*	Rate type	Bacterial cells *10^9CFU L^{-1}*	Rate constant *h^{-1}*	Normalized rate constant *(h 10^9CFU$)^{-1}$*	Normalized rate *μg (L h 10^9CFU$)^{-1}$*
0.1	mineralization	219.2	0.287	$1.31x10^{-3}$	$1.30x10^{-4}$
0.5		185.7	0.124	$6.68x10^{-4}$	$3.33x10^{-4}$
2.5		219.2	0.228	$1.04x10^{-3}$	$2.60x10^{-3}$
12.5		212.5	0.266	$1.25x10^{-3}$	$1.56x10^{-2}$
25		212.5	0.267	$1.26x10^{-3}$	$3.14x10^{-2}$
25	biotransformation	212.5	49.94	0.235	5.875

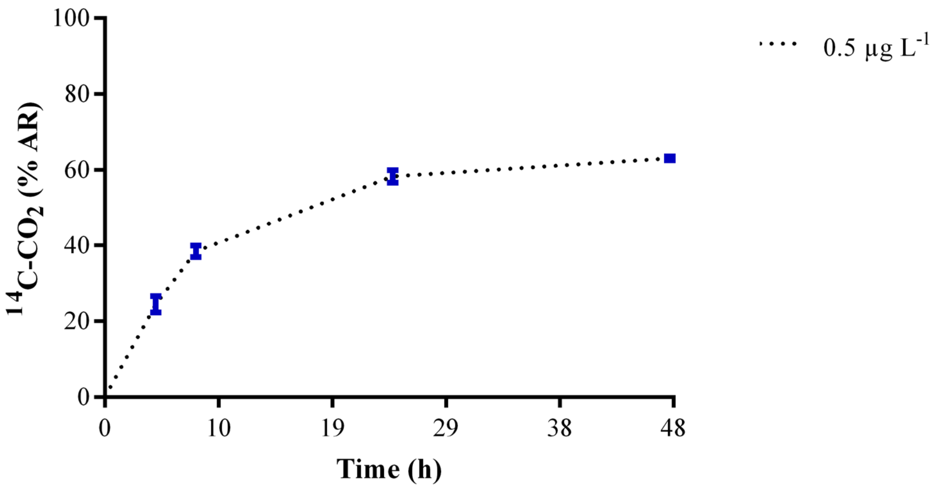

Figure S2. SMX mineralized fraction by *Microbacterium* sp BR1 after 48 h of incubation with an initial concentration of 0.5 µg L^{-1}. Mineralization occurs during 48 h of test and the observed final mineralized fraction resembles to the ones from tests incubated in a 24h term.
